# Backbonding contributions to small molecule chemisorption in a metal–organic framework with open copper(i) centers†

**DOI:** 10.1039/d0sc06038k

**Published:** 2020-12-18

**Authors:** Gregory M. Su, Han Wang, Brandon R. Barnett, Jeffrey R. Long, David Prendergast, Walter S. Drisdell

**Affiliations:** Chemical Sciences Division, Lawrence Berkeley National Laboratory Berkeley CA 94720 USA wsdrisdell@lbl.gov; The Molecular Foundry, Lawrence Berkeley National Laboratory Berkeley CA 94720 USA; Department of Chemistry, University of California, Berkeley Berkeley CA 94720 USA; Materials Sciences Division, Lawrence Berkeley National Laboratory Berkeley CA 94720 USA; Department of Chemical and Biomolecular Engineering, University of California, Berkeley Berkeley CA 94720 USA

## Abstract

Metal–organic frameworks are promising materials for applications such as gas capture, separation, and storage, due to their ability to selectively adsorb small molecules. The metal–organic framework Cu^I^-MFU-4*l*, which contains coordinatively unsaturated copper(i) centers, can engage in backbonding interactions with various small molecule guests, motivating the design of frameworks that engage in backbonding and other electronic interactions for highly efficient and selective adsorption. Here, we examine several gases expected to bind to the open copper(i) sites in Cu^I^-MFU-4*l via* different electronic interactions, including σ-donation, π-backbonding, and formal electron transfer. We show that *in situ* Cu L-edge near edge X-ray absorption fine structure (NEXAFS) spectroscopy can elucidate π-backbonding by directly probing excitations to unoccupied backbonding orbitals with Cu d-character, even for gases that participate in other dominant interactions, such as ligand-to-metal σ-donation. First-principles calculations based on density functional theory and time-dependent density functional theory additionally reveal the backbonding molecular orbitals associated with these spectroscopic transitions. The energies of the transitions correlate with the energy levels of the isolated small molecule adsorbates, and the transition intensities are proportional to the binding energies of the guest molecules within Cu^I^-MFU-4*l*. By elucidating the molecular and electronic structure origins of backbonding interactions between electron rich metal centers in metal–organic frameworks and small molecule guests, it is possible to develop guidelines for further molecular-level design of solid-state adsorbents for energy-efficient separations of relevance to industry.

## Abstract 2

*In situ* near edge X-ray absorption fine structure spectroscopy directly probes unoccupied states associated with backbonding interactions between the open metal site in a metal–organic framework and various small molecule guests.

## Graphical abstract

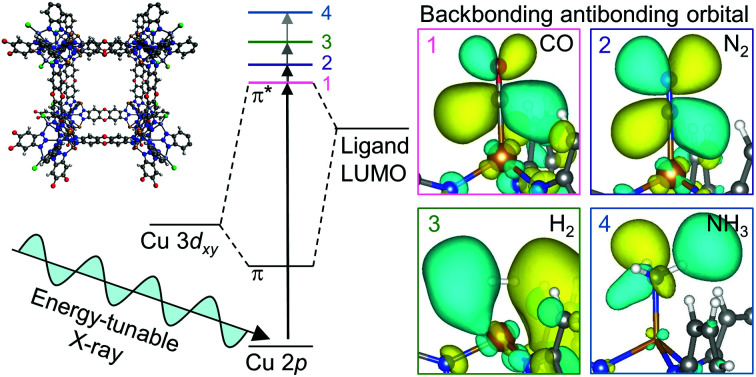

## Introduction

Metal–organic frameworks (MOFs) are a highly diverse class of crystalline, porous solids that are promising for applications including catalysis,^1,2^ molecular separations,^3–11^ hydrogen storage,^12–14^ and sensing.^15^ Frameworks featuring coordinatively-unsaturated metal sites have been studied for the adsorption of various small molecule guests,^16–18^ and electron-rich metal sites in particular can strongly bind guest molecules through charge transfer and covalent interactions.^19–21^ Metal–organic frameworks that contain open metal sites capable of engaging in π-complexation with guest molecules are especially promising for the capture of inert species and challenging separation schemes involving molecules of similar size, volatility, or polarizability.^22–24^ Particularly strong π-complexation with small molecules has been demonstrated in MOFs with the d^10^ transition metal ions Ag^+^ and Cu^+^.^25–28^ The framework Cu^I^-MFU-4*l* (Cu_2_Zn_3_Cl_2_(btdd), H_2_btdd = bis(1H-1,2,3-triazolo[4,5-*b*],[4′,5′-*i*])dibenzo[1,4]dioxin), for example, features trigonal pyramidal copper(i) sites that can readily engage in π-backbonding interactions due to the filled Cu 3d orbitals, and this material has been shown to reversibly chemisorb O_2_, N_2_, and H_2_.^29^ Notably, Cu^I^-MFU-4*l* exhibits the largest isosteric heat of adsorption for reversible H_2_ uptake of any MOF (32 kJ mol^−1^),^29^ motivating further exploration of π-backbonding as a means of achieving selective small molecule binding in these materials. The development of direct, *in situ* spectroscopic methods capable of probing backbonding interactions between metal sites and various small molecules is one key approach for identifying design rules to tune adsorption in Cu^I^-MFU-4*l* and related frameworks featuring open metal sites. Infrared (IR) spectroscopy has traditionally been used as an indirect probe of backbonding interactions with metal sites, which are manifest as red-shifts of the fundamental absorption bands of the guests involved in this interaction. However, IR spectroscopy cannot readily distinguish backbonding when it is not the dominant interaction, and therefore a more direct electronic structure probe is needed for an enhanced understanding of such interactions.

Here, we demonstrate that by using *in situ* near edge X-ray absorption fine structure (NEXAFS) spectroscopy guided by first-principles calculations, it is possible to directly probe the electronic interactions associated with the chemisorption of small molecules at the open copper(i) sites in Cu^I^-MFU-4*l*. Importantly, NEXAFS spectroscopy not only confirms backbonding interactions for classical π acceptor ligands, such as CO and N_2_, but also selectively probes weaker backbonding interactions of the σ-donating adsorbates C_2_H_4_ and NH_3_. The case of ammonia is particularly remarkable, as its interaction with copper is dominated by electron donation. However, NEXAFS spectroscopy of ammonia-bound Cu^I^-MFU-4*l* reveals the presence of unoccupied states with Cu d-character and weak backbonding, an interaction that is difficult to probe with other spectroscopic techniques. Additionally, we find that a side-on orientation of adsorbed O_2_ and triplet configuration of the O_2_-bound Cu^I^-MFU-4*l* complex is energetically favorable, contributing to a binding mechanism that resembles Cu^I^-MFU-4*l*'s interaction with H_2_, N_2_, or CO but with a greater degree of electron transfer. *In situ* NEXAFS L-edge spectroscopy therefore enables a detailed assessment of metal–adsorbate bonding interactions, even in the case of metal ions with a filled valence d-manifold.

## Experimental

### Materials and sample preparation

The synthesis of Cu^I^-MFU-4*l* was adapted from the procedure from Denysenko *et al.*^29^ and carried out as previously reported.^30^ Cu^I^-MFU-4*l* was deposited on X-ray transparent 150 nm thick, 2.0 mm × 2.0 mm silicon nitride windows supported by a silicon frame (Silson Ltd.). To improve adhesion of the framework particles to the substrate, polystyrene (*M*_w_ = 350 kg mol^−1^, *M*_n_ = 170 kg mol^−1^, Sigma-Aldrich) was dissolved in toluene at a concentration of 20 mg mL^−1^ and spin coated on top of the silicon nitride windows (2000 rpm, 40 s) prior to framework deposition, to form a thin polystyrene film. Polystyrene is useful for this purpose partly because it exhibits minimal absorption near the Cu L-edge. As-synthesized Cu^I^-MFU-4*l* was separately suspended in *n*-hexane in an argon-filled glovebox, sealed in a borosilicate vial, removed from the glovebox and sonicated for 5 min. The vial was then brought back into the glovebox, and the framework was drop-cast atop the polystyrene-coated silicon nitride window. After evaporation of the hexanes, the window was heated *in vacuo* (∼ 50 mTorr) at 80 °C for at least 2 h. Samples were then sealed in vials and transferred to a nitrogen glove box where they were loaded into a custom-built gas cell and sealed before bringing to the beamline for NEXAFS measurements.

### NEXAFS measurements

Soft X-ray NEXAFS spectroscopy measurements were carried out at room temperature at energies near the Cu L-edge at bending magnet beamline 6.3.2 (10^11^ photons per s) at the Advanced Light Source at Lawrence Berkeley National Laboratory. Gas dosing experiments were carried out with a previously described custom-built gas cell.^31–33^ The beamline X-ray energy was calibrated to the edge step of a Cu filter, which was set to 1.3293 nm (932.7 eV). Once attached to the beamline, the sample was pumped and kept at high vacuum (∼10^−7^ Torr) for at least 30 min to activate the framework before any spectra were collected. The transmission NEXAFS spectra of the activated material was then collected before exposure to any gas. Following collection of NEXAFS spectra of the activated framework, H_2_, N_2_, CO, O_2_, NH_3_, or C_2_H_4_ gas was slowly introduced in the gas cell, and spectra were collected *in situ* at varying pressures up to 1 bar. After dosing with each gas, the framework sample was evacuated in the gas cell using high vacuum and a final spectrum was measured. A bare polystyrene-coated silicon nitride window was used for background correction. For normalization, a line was regressed to the pre-edge region and a polynomial regressed to the post-edge region using the Athena software package.^34^ The sample was not moved during data collection to minimize any effects arising from inhomogeneity in material thickness or distribution. Beam-induced damage to the sample is minimal on the timescale of the measurement because of the relatively low X-ray flux per unit area on the sample, due to the position of the gas cell ∼1 m past the X-ray beam focal point.^32^ Multiple NEXAFS scans taken on the same spot before gas dosing show nearly identical spectra (ESI Fig. S1†), confirming minimal beam damage.

### Computational methods

An 86-atom cluster with only one Cu atom at each ligand-MOF juncture was generated from the periodic framework structure by replacing the btdd^2−^ ligands with terminal benzotriazolate groups (ESI Fig. S2†). Copper and nitrogen atoms in the MOF cluster and atoms on the guest molecules were calculated using the def2-SVPD^35^ basis-set, while the def2-SVP basis set was used for the remaining atoms. X-ray absorption spectra were calculated using the restricted energy window (REW) linear-response time-dependent density-functional theory (LR-TDDFT) method implemented within Q-Chem. All systems were treated as singlets, except the O_2_-bound cluster, which was treated as a triplet because the calculated energy of the triplet configuration was noticeably lower than the singlet configuration, as discussed later. The M06-2X hybrid functional was used for both structure relaxations and LR-TDDFT calculations. The M06-2X functional is a global hybrid meta-GGA functional with 54% Hartree–Fock (HF) exchange. Previous work has shown that a large amount of HF exchange in the short and mid-range is necessary for accurate NEXAFS calculations using TDDFT.^36^ M06-2X has a relatively large HF exchange and performs well for the systems studied here. Each calculated transition was convoluted with a Gaussian line shape (*σ* = 1.0 eV) to produce a continuous spectrum. Since spin–orbit coupling effects are not included in our LR-TDDFT calculation, the calculated spectrum is for one representative 2p core orbital, which we designate as 2p_3/2_ or the L_3_-edge. The L_2_ edge, arising from 2p_1/2_ excitations, is reproduced approximately by applying the atomic 2p spin–orbit splitting of 20.0 eV to the original spectrum, with a reduction to 50% intensity to reflect that there are only two electrons in the 2p_1/2_ orbitals *vs.* four in the 2p_3/2_ orbitals. The first LR-TDDFT root energy was approximated with the maximum overlap method (MOM)^37^ by moving one electron from a Cu 2p orbital to the unoccupied molecular orbital indicated by the LR-TDDFT root. To assign a common energy scale to all simulated spectra, the first LR-TDDFT root energy was aligned to the energy difference between the MOM calculation and the ground state calculation (Table S1†). The resulting energy scale was systematically shifted compared to the experimental spectra, so simulated spectra were shifted by −6.7 eV to compensate.

## Results and discussion

NEXAFS is a spectroscopic technique that broadly reflects core-level transitions to unoccupied states and can therefore reveal *in situ* electronic perturbations of the d manifold in transition metals. For 3d metals, K-edge NEXAFS primarily probes 1s → 4p transitions, while L_2,3_-edge NEXAFS probes 2p → 3d transitions. Metal L-edge NEXAFS spectroscopy directly probes the valence d manifold, including vacant hybrid states that have 3d character, and as a result can reveal backbonding interactions responsible for small molecule binding to unsaturated 3d metal centers. The specificity of 3d metal L-edge NEXAFS spectroscopy to a specific metal element, and to electronic states with 3d character, allows for quantitative characterization of backbonding interactions in complex framework systems, such as mixed-metal MOFs^38^ or MOFs with functional groups tethered to the open metal sites that show cooperative adsorption behavior.^33,39^ For a system with a nominally filled d manifold, such as copper(i) (4s^0^3d^10^) in Cu^I^-MFU-4*l*, NEXAFS probes transitions to antibonding states with Cu 3d character that form from such backbonding interactions. A new feature in Cu L-edge NEXAFS upon gas dosing is therefore a signature of a backbonding interaction.^40^

We studied several gases expected to have varying interactions with Cu^I^-MFU-4*l* to develop predictive spectroscopic capabilities that can uniquely decipher backbonding interactions involving small molecules and metal d orbitals, even for unsaturated metal centers with a filled valence d-shell. In order to investigate differences among ligand interactions dominated by π-backbonding, H_2_, N_2_, and CO were chosen as initial probe molecules. Likewise, ammonia, ethylene, and O_2_ were chosen to study metal–adsorbate interactions wherein other bonding interactions are expected to dominate. For example, NH_3_ has an electron lone pair that donates into the 4s/4p orbitals of Cu^+^ and has no unoccupied states with energies similar to the Cu^+^ 3d orbitals to allow for significant backbonding. Ethylene (C_2_H_4_) is expected to donate electron density to the 4s/4p orbitals of Cu^I^, similarly to ammonia, but also to accept significant electron back-donation into its π* orbital. Dioxygen should initiate an electron transfer and effectively oxidize the copper center. A detailed understanding of these backbonding and electron transfer interactions is of interest for guiding the design of frameworks for next-generation gas separations and storage.

Importantly, the LUMO energy of the isolated small molecule ligands is reduced when the small molecule is in close proximity to Cu^+^. This electrostatic shift must be considered when building molecular orbital diagrams for these coordination complexes, and is not often discussed in detail. For the molecular orbital diagrams presented in Fig. 2, 5, and ESI Fig. S11,† the calculated LUMO energies of the free ligands are shown together with the electrostatic shift that occurs near the open copper(i) site.

### Correlation between NEXAFS transition and isolated guest LUMO energies

**Fig. 1:**
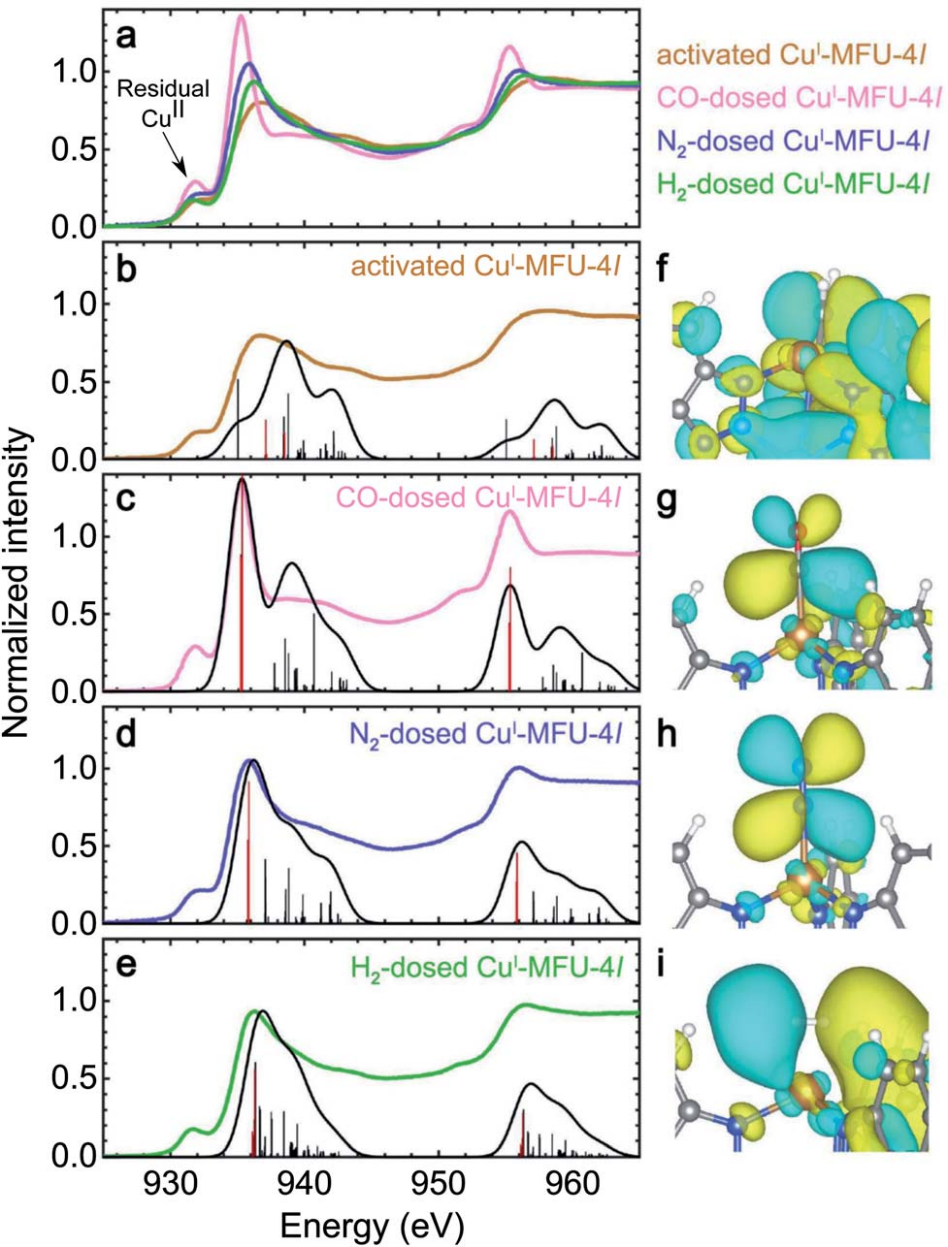
(a) Experimental room temperature Cu L-edge NEXAFS spectra of bare Cu^I^-MFU-4*l* and Cu^I^-MFU-4*l* dosed *in situ* with 0.25 mbar CO, 1000 mbar N_2_, or 1000 mbar H_2_. The pre-edge feature near 931.8 eV is attributed to residual copper(ii) in the framework. Individual experimental and simulated spectra are plotted together in (b) to (e) for the bare framework and Cu^I^-MFU-4*l* dosed with CO, N_2_, and H_2_, respectively. Vertical lines indicate individual transitions, while the solid trace is the average Gaussian-convoluted simulated spectrum. The thick red lines correspond to major transitions from the core 2p orbitals to final states between 935 and 936 eV. The molecular orbitals of these final states are shown on the right for (f) bare Cu^I^-MFU-4*l* and the framework dosed with (g) CO, (h) N_2_, and (i) H_2_. In (g)–(i), the final states correspond to unoccupied, antibonding orbitals generated upon π-backbonding between copper(i) and the given guest.

The experimental and calculated Cu L-edge NEXAFS spectra of bare Cu^I^-MFU-4*l* and Cu^I^-MFU-4*l* in the presence of CO, N_2_, and H_2_ are shown in Fig. 1a–e, along with the calculated wave functions (molecular orbitals) of representative final states in Fig. 1f–i. All experimental spectra possess a small pre-edge feature at 931.5 eV, which is attributed to the presence of copper(ii), presumably due to incomplete autoreduction of Cu^II^-MFU-4*l* to Cu^I^-MFU-4*l*.^29^ This pre-edge feature is not present in the simulations because idealized clusters containing no residual copper(ii) were used for the calculations. Upon dosing with each gas, a new feature emerges at 935.3, 935.9, and 936.3 eV for CO-, N_2_-, and H_2_-dosed samples, respectively. Spectra computed using LR-TDDFT clearly reproduce this feature and its relative energy among the three gases, although the match between the calculated and experimental spectra for the bare framework is not as good. This result is likely due to the finite cluster size used in the calculations, which does not capture the extended, delocalized states present in the bulk framework structure.

The simulated electron density distributions associated with the new spectral features upon dosing with H_2_, N_2_, or CO confirm that Cu^I^-MFU-4*l* engages in π-backbonding with these small molecules, and this includes adsorbates like N_2_ that are generally not considered strong π-acceptors. This underscores the backbonding strength of the open copper(i) sites in Cu^I^-MFU-4*l* relative to metal centers with oxidation states of +2 or greater typically found in MOFs. In each case, the feature arises from a core-level transition to an unoccupied, antibonding molecular orbital generated upon π-backbonding between copper(i) and the adsorbed gas, with the peak energy correlating with the relative LUMO energy of the isolated ligand. For example, the electron density distribution in Fig. 1g shows the final state near 935.3 eV for Cu^I^-MFU-4*l* dosed with CO and confirms π symmetry. Antibonding character is verified by the nodal surface, and this antibonding state has clear Cu 3d and CO π* character. The interaction of Cu^I^-MFU-4*l* with N_2_ is similar (Fig. 1h), confirming a π-backbonding interaction. Hydrogen also engages in π-backbonding with copper(i), but unlike CO and N_2_, electron density from the Cu 3d orbital donates into the H_2_ σ* orbital (Fig. 1i). H_2_ adsorbs in a side-on configuration such that the backbonding interaction has π symmetry, even though it is formed with a σ orbital. Furthermore, simulated NEXAFS spectra as a function of Cu–H_2_ distance supports that the Cu–H_2_ distance is close to 1.7 Å (ESI Fig. S3†), in agreement with experimental measurements and previous DFT calculations.^41^

**Fig. 2:**
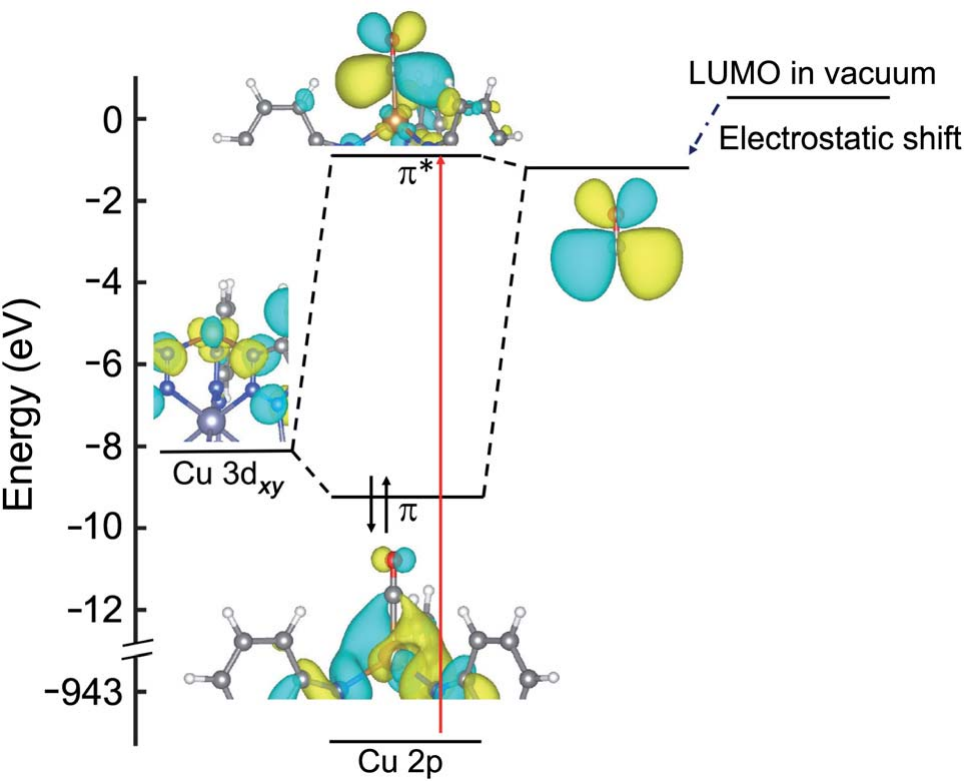
Molecular orbital diagram of Cu^I^-MFU-4*l* with adsorbed CO. The energies of the HOMO (not shown) and LUMO of the isolated CO ligand are reduced when in close proximity (∼2 Å) to copper(i), due to the strong electric field of the metal center. The red arrow indicates the transition probed by NEXAFS that appears as a new peak at 936.9 eV as shown in Fig. 1c.

An orbital diagram representing the interaction between the framework and adsorbed gas can be constructed from a simplified two level model that includes a Cu 3d orbital and the ligand LUMO (see Fig. 2 for a representative diagram for adsorbed CO). These two orbitals couple to generate one bonding and one antibonding state, and the backbonding peak that emerges in the Cu L-edge NEXAFS spectra upon ligand adsorption represents a transition of a core-level Cu 2p electron to this unoccupied, antibonding orbital. As shown in ESI Table S2 and ESI Fig. S4,† the higher the relative LUMO energy of the free adsorbate, the higher the energy of the antibonding orbital generated upon π-backbonding, which dictates the energy of the corresponding NEXAFS transition.

The intensity of the backbonding peak scales with the strength of the backbonding interaction, *i.e.*, the percentage of Cu 3d character in the antibonding orbital. For example, previous work has shown that the peak intensity of the L-edge spectra of copper(ii) complexes can be used to estimate the copper character in a system with a half-occupied HOMO.^42^ The intensity will accordingly also scale with the fraction of bound copper(i) sites, and this effect is readily seen by examining peak height as a function of gas pressure. For example, dosing Cu^I^-MFU-4*l* with increasing pressures of N_2_ or H_2_ up to 1 bar results in an increase in the intensity of the corresponding backbonding peak for each guest, reflecting increasing occupation of the open copper(i) coordination sites (ESI Fig. S5†). Indeed, adsorption isotherms collected for N_2_ and H_2_ at 25 °C indicate that even at 1 bar, only approximately two thirds and half of the copper(i) sites are occupied by each gas, respectively (ESI Fig. S6†). However, in the case of CO, increasing the pressure from 0.15 to 0.25 mbar has no effect on the resulting NEXAFS backbonding peak intensity, suggesting that the available copper(i) sites are fully saturated with the gas, even at these low pressures. These data indicate strong binding of CO in Cu^I^-MFU-4*l*, as further supported by CO adsorption data collected at 25 °C (ESI Fig. S7†), which show steep uptake of CO and saturation at <0.1 mbar.

### Correlating peak intensity with binding strength

Although factors such as copper site coverage and the absorbance of X-rays by the free gas will influence experimental peak intensities, high-level simulations enable the direct correlation of peak intensities to the binding energy of a given guest in Cu^I^-MFU-4*l*. In particular, the Hamiltonian matrix of the simplified two-level system discussed here can be expressed as1
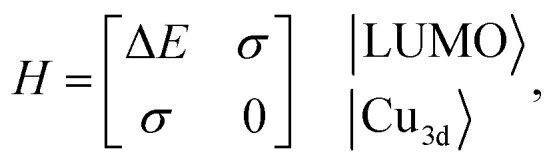
where Δ*E* is the energy difference between the LUMO level of the gas molecule and Cu 3d orbital and *σ* is the so called hopping integral,^43,44^ which represents the interaction between Cu 3d orbitals and the ligand LUMO. The energy of the antibonding orbital formed between a Cu 3d orbital and ligand LUMO can be obtained by solving the two-level Hamiltonian model,2
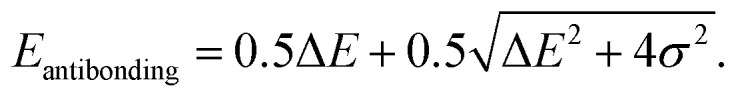


Since the antibonding orbital is the final state of the NEXAFS excitation, the antibonding orbital energy determines the NEXAFS peak position. Due to the weak interaction between copper and the adsorbed molecule, *σ* is expected to be much smaller than Δ*E* and thus the antibonding orbital energy is primarily determined by Δ*E*, which explains the correlation between the NEXAFS peak position and the isolated ligand LUMO energy (ESI Fig. S4†).

The contribution of the Cu 3d orbital to the antibonding orbital wavefunction can be expressed as3
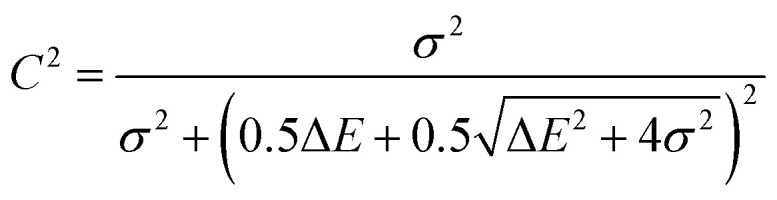


The binding energy, *E*_b_, is the energy difference between the bonding orbital energy and the total energy of the system before forming the bond,4
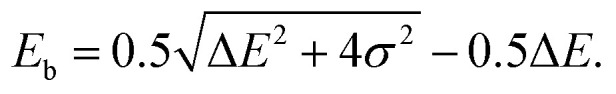


Assuming that *σ* is much smaller than the energy difference, Δ*E*, then5
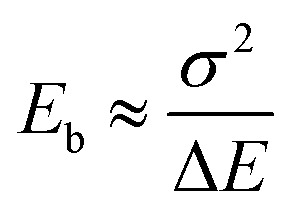
and6
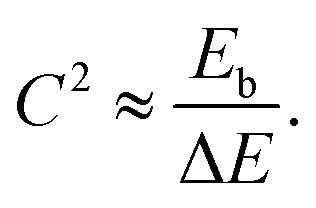


This relationship indicates that for guest molecular orbitals interacting with a given Cu 3d orbital, the intensity of the associated NEXAFS peak will be proportional to the binding energy of the guest molecule. The binding energy and calculated *C*^2^ value of the molecules studied here are shown in ESI Table S2.† Our model therefore shows that the binding energy of a small molecule at a copper(i) site within a MOF can be predicted by the Cu L-edge NEXAFS peak intensity.

### Weak backbonding interactions with NH_3_

**Fig. 3:**
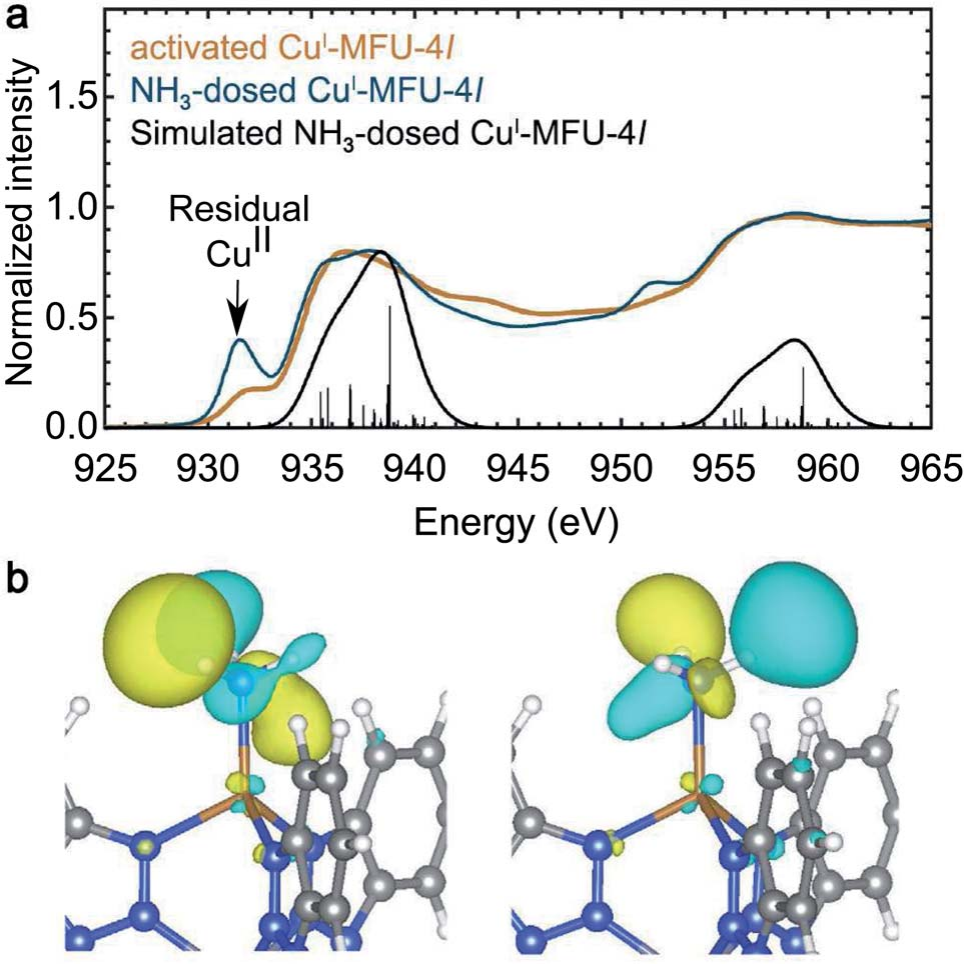
(a) Room temperature NEXAFS spectra of bare Cu^I^-MFU-4*l* (yellow) and Cu^I^-MFU-4*l* dosed *in situ* with 50 mbar NH_3_ (blue). The pre-edge feature near 931.8 eV is attributed to residual copper(ii) in the framework, and its increase in intensity upon NH_3_ dosing suggests NH_3_ also binds to these sites. The Gaussian-convoluted simulated spectrum of NH_3_-dosed Cu^I^-MFU-4*l* is shown in black. Vertical lines indicate individual transitions. (b) Electron density distribution plots of final states contributing to the feature near 938 eV. The Cu atom radius is reduced to show the 3d orbitals.

Ammonia (NH_3_) is expected to interact with copper(i) primarily *via* σ donation of its electron lone pair into the hybridized 4s/4p states of the metal center, as shown in ESI Fig. S11.† However, NEXAFS L-edge spectroscopy is insensitive to unoccupied states with Cu 4p character, instead revealing weak backbonding interactions that are often overlooked. Although π backbonding from NH_3_ is not expected to substantially perturb the Cu 3d manifold, the room temperature *in situ* NEXAFS spectrum of Cu^I^-MFU-4*l* dosed with 50 mbar of NH_3_ exhibits changes in the main L_3_-edge feature between 935 and 939 eV relative to the bare framework, suggesting the presence of new unoccupied states with d-character. In particular, the bare framework displays a peak at 936 eV and a weaker feature at 938 eV, but after dosing with NH_3_, the feature at 938 eV increases in intensity relative to the lower energy feature. The simulated NEXAFS spectrum of NH_3_-dosed Cu^I^-MFU-4*l* reproduces these peak positions and relative intensities (Fig. 3a). Analysis of the states contributing to these peaks reveals that the increased intensity at 938 eV results from excitations into antibonding orbitals with a small amount of Cu 3d character, associated with π-backbonding into a NH_3_ σ* orbital. Therefore, NEXAFS spectroscopy functions as a reliable probe for even these weak back-donation interactions. The electronic states that contribute most to the peak at 938 eV are shown in Fig. 3b. These states clearly display π character in the interaction between the Cu 3d and NH_3_ molecular orbitals, including the LUMO+1 and LUMO+2 of ammonia. Small amounts of LUMO+3 and LUMO+4 character are visible as well (ESI Table S2†). The absence of NH_3_ LUMO character in the states that contribute to the NEXAFS peak reveals that the LUMO+1 and LUMO+2 orbitals of ammonia couple more strongly than the LUMO orbital with the open copper(i) centers. Ultimately, these data reveal that *in situ* NEXAFS spectroscopy, coupled with simulations, is sensitive to weak backbonding interactions even in the presence of stronger electronic interactions that do not involve the metal d manifold. Theory-driven X-ray absorption spectroscopy is therefore a powerful tool for deconvoluting complex metal–ligand interactions.

Finally, it is worth noting that the copper(ii) pre-edge feature of Cu^I^-MFU-4*l* at 931.5 eV increases in intensity with increasing NH_3_ pressure, and this feature retains its elevated intensity even after the sample is exposed to high vacuum (ESI Fig. S5†). This may be due to ammonia binding more strongly to the more Lewis acidic Cu^2+^ sites in the framework. However, further investigation is needed to fully understand if ammonia adsorption at residual copper(ii) sites involves backbonding or other electronic interactions.

### Strong backbonding interactions with ethylene

**Fig. 4:**
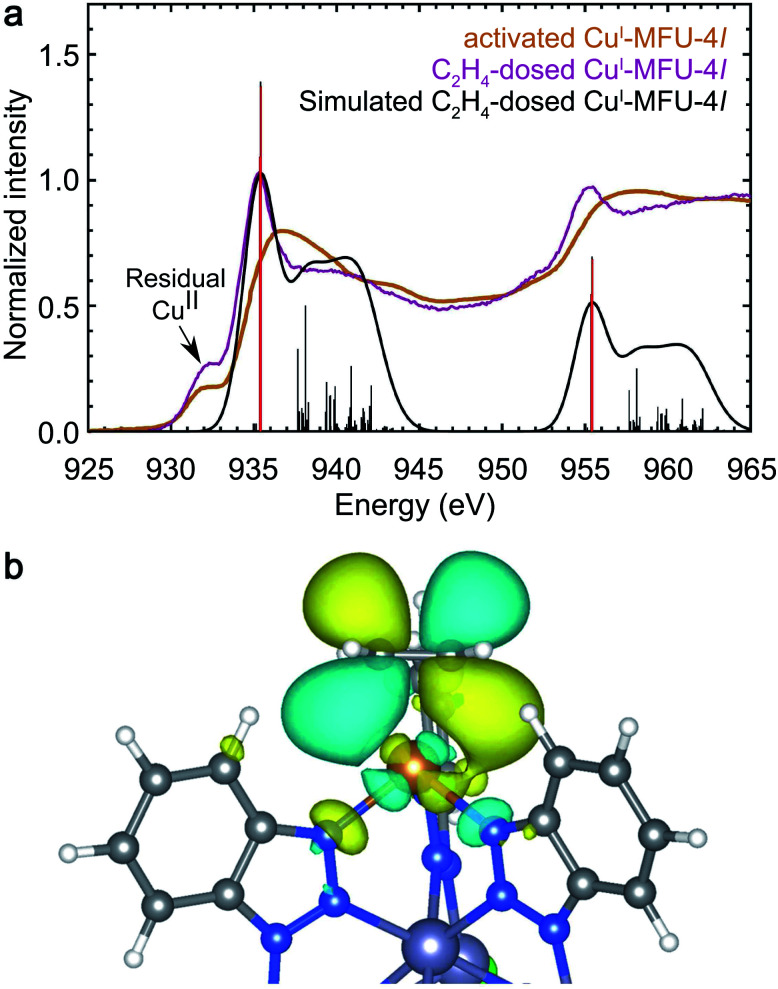
(a) Experimental room temperature NEXAFS spectra of bare Cu^I^-MFU-4*l* (yellow) and Cu^I^-MFU-4*l* dosed *in situ* with 1 mbar C_2_H_4_ (purple). The pre-edge feature near 931.8 eV is attributed to residual copper(ii) in the framework. The Gaussian-convoluted simulated NEXAFS spectrum of C_2_H_4_-dosed Cu^I^-MFU-4*l* is shown in black. Vertical lines indicate individual transitions. (b) An electron density distribution plot of a representative final state contributing to the feature at 935.4 eV.

Ethylene (C_2_H_4_) is expected to interact with Cu^I^-MFU-4*l* through backdonation as well as significant forward donation of electron density from its filled π HOMO orbital into available 4s or 4p orbitals on copper(i). *In situ* NEXAFS reveals the emergence of a strong backbonding peak upon dosing with ethylene, similar to H_2_, N_2_, and CO. In particular, backbonding from the Cu d_*xz*_ or d_*yz*_ orbital into the unoccupied π* LUMO of ethylene (ESI Table S2†) is expected to occur. Fig. 4 reveals the appearance of a new NEXAFS peak upon ethylene dosing, which confirms the presence of an unoccupied orbital with significant Cu d-character. The simulated spectrum of C_2_H_4_-dosed Cu^I^-MFU-4*l* reproduces the new peak at 935.4 eV and an electron density distribution plot corresponding to the final state confirms that what is being probed is an antibonding orbital associated with the ethylene π-backbonding interaction (Fig. 4).

### Covalency in O_2_-bound Cu^I^-MFU-4*l*

**Fig. 5:**
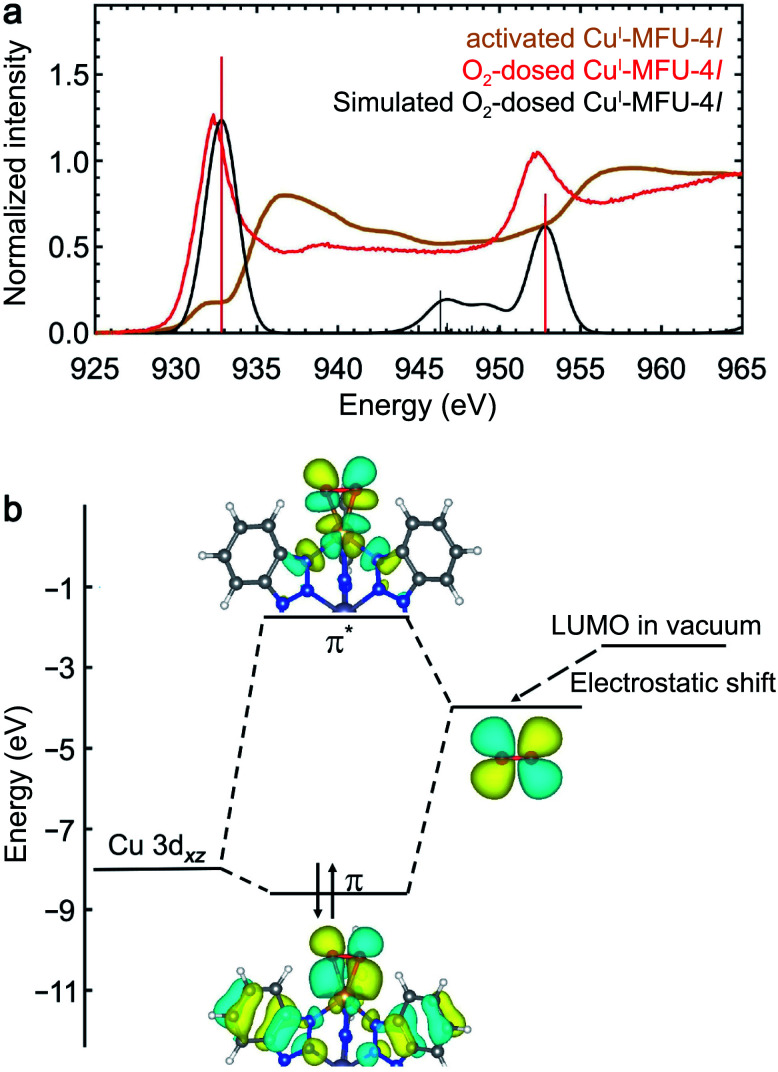
(a) Experimental room temperature NEXAFS spectra of bare Cu^I^-MFU-4*l* (yellow) and Cu^I^-MFU-4*l* dosed *in situ* with 1000 mbar O_2_ (red). The Gaussian-convoluted simulated NEXAFS spectrum of O_2_-dosed Cu^I^-MFU-4*l* is shown in black. Vertical lines indicate individual transitions. (b) Molecular orbital diagram depicting the interaction between copper(i) and O_2_.

The NEXAFS spectrum of O_2_-dosed Cu^I^-MFU-4*l* features a new, intense peak at 932.4 eV relative to the spectrum of the bare framework (Fig. 5) and is similar to reported spectra of CuO,^45^ suggesting the Cu^+^ centers are oxidized to Cu^2+^ upon O_2_ adsorption. Natural bond orbital (NBO) analysis reveals that the charge on Cu increases from 0.889 to 1.39*e* upon O_2_ binding, supporting strong covalency and significant electron transfer from the copper ion to O_2_ (ESI Table S3†). Although this redox behavior is distinct, the Cu^+^–O_2_ interaction shows similar features to the π acceptor ligand molecules. However, given that the LUMO of isolated O_2_ (−1.58 eV) is significantly lower in energy compared to the other small molecule ligands studied (ESI Fig. S4†), this energy match is also expected to lead to strong coupling and significant electron transfer from copper(i) to O_2_. Correspondingly, the new peak that appears upon dosing Cu^I^-MFU-4*l* with O_2_ is significantly lower in energy relative to the backbonding peaks that appear in the presence of H_2_, N_2_, CO, or C_2_H_4_.

The NEXAFS data and simulations reveal covalency between the copper(i) centers and adsorbed O_2_ and donation of electron density from Cu 3d orbitals into the O_2_ π* orbital. A molecule of O_2_ adsorbed at a copper center in Cu^I^-MFU-4*l* can adopt either a bent end-on or side-on orientation. Despite the covalency of the bond, the O_2_ remains in its triplet state, as determined by its lower energy, because the binding between O_2_ and Cu^+^ is not strong enough to compensate for the large energy difference between singlet and triplet O_2_ (1.6 eV). The triplet side-on configuration is lower in energy than the triplet bent end-on configuration by ∼0.23 eV (ESI Fig. S12†). As shown in ESI Fig. S12(c–f),† the simulated NEXAFS spectra of an O_2_-bound Cu^I^-MFU-4*l* complex in a triplet state and a side-on O_2_ orientation best reproduces the most prominent experimental NEXAFS features, supporting that this is an accurate representation of the real sample. It is worth noting that the LR-TDDFT simulated NEXAFS of side-on triplet O_2_ is misaligned by ∼5 eV relative to the other singlet state adsorbate-bound Cu^I^-MFU-4*l* complexes. This can be addressed with the MOM shifting method by using the excited-state charge density to estimate the energy difference between the ground state and the excited state that matches the orbital character of the first LR-TDDFT root. The corresponding energy shift is shown in ESI Fig. S13.† Electron density distribution plots of the bonding and anti-bonding states associated with the Cu^I^–O_2_ interaction reveal noticeable Cu d-character and O_2_ p-character (Fig. 5). This interaction mimics the character of the interaction between Cu^I^-MFU-4*l* and H_2_, N_2_, or CO, but with a much greater degree of electron transfer.

## Conclusions

The foregoing results reveal that *in situ* NEXAFS spectroscopy coupled with first-principles calculations is a powerful approach for directly interrogating the electronic structure of metal ions in MOFs, including metal centers with a filled d-manifold. In particular, we have illustrated the sensitivity of this approach to backbonding interactions between metal d-orbitals and antibonding orbitals of small molecule guests, even in cases where other electronic interactions, such as σ-donation, dominate. Interactions between the copper(i) sites in Cu^I^-MFU-4*l* and adsorbed H_2_, N_2_, or CO are dominated by π-backbonding involving transfer of electron density from the metal to an unoccupied orbital of the guest molecule. This interaction forms both bonding and antibonding states, and Cu L-edge NEXAFS directly probes the transition into the resulting antibonding molecular orbital, which is manifest as a distinct NEXAFS peak upon ligand adsorption. The energy of the backbonding peak correlates with the isolated ligand LUMO energy, and electron density distribution plots based on DFT calculations confirm this peak represents an antibonding π-backbonding state. Ammonia, by contrast, interacts with Cu^I^-MFU-4*l* primarily through electron donation into Cu 4s/4p orbitals, but the NEXAFS spectrum of NH_3_-dosed Cu^I^-MFU-4*l* indicates that weak backbonding interactions with Cu d-character also occur. Ethylene adsorption involves both strong electron donation and π-backbonding, while O_2_-dosed Cu^I^-MFU-4*l* exhibits spectral features similar to CuO, suggesting oxidation of the copper centers. NBO analysis shows significant charge transfer from copper to O_2_, and calculated molecular orbitals reveal that this interaction mimics that of π-acceptor ligands, albeit with a larger degree of electron transfer. Finally, the intensity of the backbonding peak that emerges upon ligand adsorption reflects the magnitude of the ligand binding energy in Cu^I^-MFU-4*l*.

The distinct spectral response for each of the gases studied highlights the utility of theory-driven NEXAFS spectroscopy as a direct probe of electronic structure and key interactions between frameworks with open metal sites and small molecules. As a result of the selectivity of NEXAFS spectroscopy for specific electronic states, complex adsorption interactions can be understood in detail using this technique. This level of detail is useful for materials discovery efforts, in which NEXAFS spectroscopy can be employed to test candidate materials and guide synthetic approaches for tailoring specific adsorption interactions with target molecules.

## Conflicts of interest

There are no conflicts to declare.

## Supplementary Material

SC-012-D0SC06038K-s001
