# Supplementary material for: Backbonding contributions to small molecule chemisorption in a metal–organic framework with open copper(i) centers
Source: Chem Sci. 2020 Dec 18;12(6):2156–64. doi: 10.1039/d0sc06038k (PMC8179296; doi:10.1039/d0sc06038k)
Supplement: SC-012-D0SC06038K-s001 [file SC-012-D0SC06038K-s001.pdf]

Electronic Supplementary Material (ESI) for Chemical Science.
This journal is © The Royal Society of Chemistry 2020

Supporting Information: Backbonding Contributions to Small Molecule Chemisorption in a Metal–Organic Framework with Open Copper(I) Centers

Gregory M. Su,[†,⊥] Han Wang,[‡,⊥] Brandon R. Barnett,[¶,§] Jeffrey R. Long,[¶,‖,§] David Prendergast,[‡] and Walter S. Drisdell[*,†]

†*Chemical Sciences Division, Lawrence Berkeley National Laboratory, Berkeley, CA 94720, USA.*

‡*The Molecular Foundry, Lawrence Berkeley National Laboratory, Berkeley, CA 94720, USA.*

¶*Department of Chemistry, University of California, Berkeley, Berkeley, CA 94720, USA.*

§*Materials Science Division, Lawrence Berkeley National Laboratory, Berkeley, CA 94720, USA.*

‖*Department of Chemical and Biomolecular Engineering, University of California, Berkeley, Berkeley, CA 94720, USA.*

⊥*These authors contributed equally to this work*

E-mail: wsdrisdell@lbl.gov

Beam damage

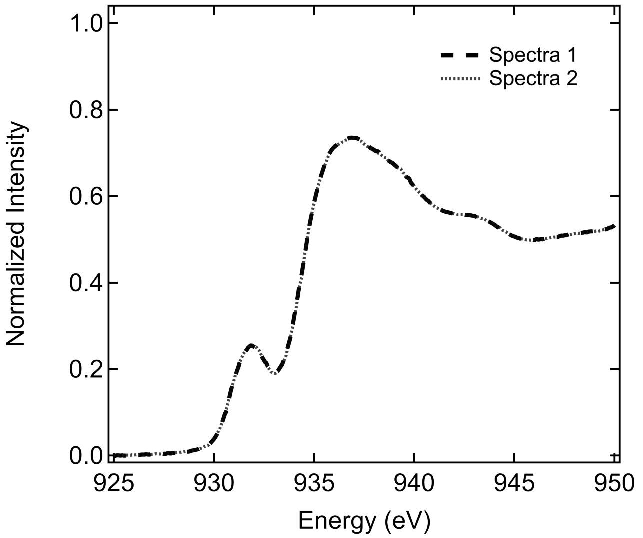

Figure S1: Two consecutive NEXAFS measurements taken on the same spot of activated (bare) Cu^{I}-MFU-4*l* reveal nearly identical spectra, suggesting minimal beam damage.

Cu^{I}-MFU-4*l* geometry optimization and truncated cluster for NEXAFS calculations

Simulated NEXAFS spectra depend strongly on the MOF structure. In Cu^{I}-MFU-4*l*, the Cu–ligand distance and angle will determines the corresponding peak position and line shape in a given spectrum. The fine structure of the calculated spectra depends on both the functional used and the MOF cluster structure. The functional used also influences geometry relaxation results. We tested different functionals, including B3LYP, PBE0, M06-2X, and ω98b-v. M06-2X gave optimal results for relaxing the MOF geometry and calculating the NEXAFS spectra with LR-TDDFT.

The full unit cell of bare Cu^{I}-MFU-4*l* has over 600 atoms and a lattice constant of 31.22 Å at room temperature, which results in calculations that are computationally expensive. To reduce the computational difficulty, geometry relaxations and NEXAFS simulations were

performed on a representative cluster taken from the bulk MOF structure, which contains one Cu center, as shown in Figure S2.

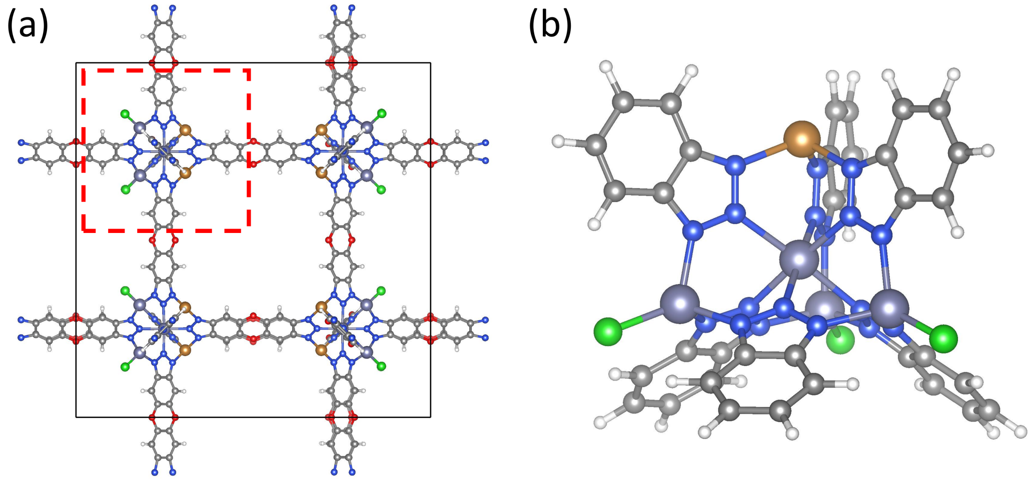

Figure S2: The unit cell of Cu^{I}-MFU-4l is shown in (a) and the cluster structure cut from the red cubic region of the periodic MOF structure used for simulations is shown in (b). The grey, white, red, blue, orange, cyan and green spheres represent C, H, O, N, Cu, Zn, and Cl atoms, respectively.

Table S1: The energy of the first LR-TDDFT root, the electron-hole pair used in the MOM calculation, and the corresponding total energy difference between the MOM calculation and the ground state calculation, respectively. α **and** β **represent the spin up and spin down channels, respectively.**

	MOF	O_2 side-on (T_1)	O_2 end-on (T_1)	CO	N_2	H_2	C_2H_4	NH_3
$\epsilon^{1st\,root}_{LR-TDDFT}$ (eV)	937.4981	930.1682	936.4594	938.1185	938.6164	939.3351	937.5931	938.7795
MOM	$\alpha 28 \rightarrow 290$	$\beta 28 \rightarrow 292$	$\beta 28 \rightarrow 291$	$\alpha 28 \rightarrow 293$	$\alpha 28 \rightarrow 295$	$\alpha 28 \rightarrow 287$	$\alpha 28 \rightarrow 294$	$\alpha 27 \rightarrow 295$
$E_{MOM} - E_{GS}$ (eV)	941.6636	939.4004	940.2748	941.7865	942.3894	943.1808	941.8823	942.1562

Predicting the Cu–H_2 distance

Figure S3 presents the experimental H_2 dosed spectrum of Cu^{I}-MFU-4l with six different computed spectra wherein the adsorbed H_2 distance decreases from 2 to 1.5 Å. With decreasing distance between H_2 and the metal site, an additional peak clearly grows in at 938 eV. By analyzing the contributing orbital, we find that it originates from the LUMO+1 orbital of H_2. Since the H_2 LUMO+1 is more localized compared with the LUMO state, strong

coupling occurs only at short Cu–H_2 distances. Since the peak at 938 eV is not visible in the experimental spectrum, the Cu–H_2 distance is expected to be more than 1.7 Å.

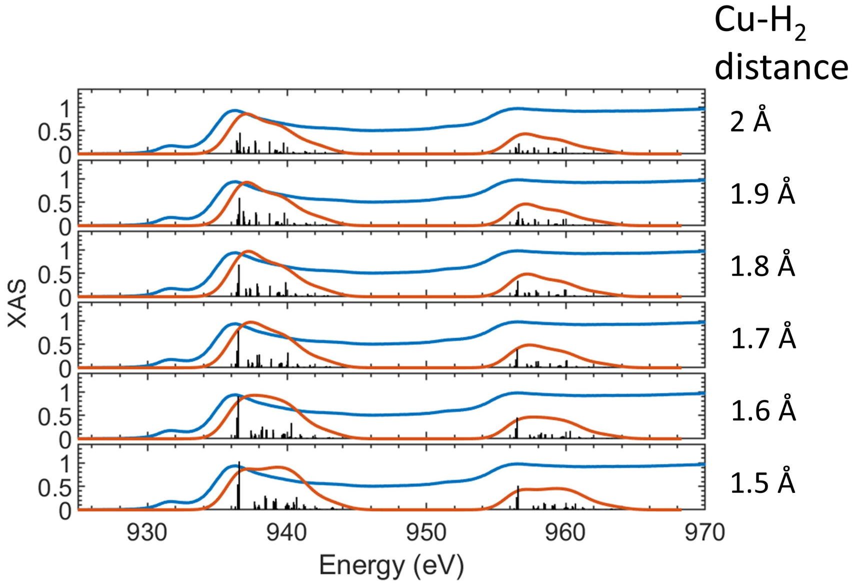

Figure S3: Experimental (blue) and simulated (red) NEXAFS spectra for H_2 adsorbed in Cu^{I}-MFU-4*l*. The simulated spectra were generated by varying the Cu–H_2 distance from 1.5 to 2 Å. A second peak at approximately 938 eV increases in intensity as the Cu–H_2 distance decreases.

Correlating NEXAFS transition and isolated guest LUMO energies

The NEXAFS backbonding peak intensity depends largely on the binding energy of a given adsorbate at the open copper site. Since the coupling between ligand and Cu varies with different adsorbates, the peak position is highly dependent on the LUMO energy of the isolated ligand molecule (Figure S4).

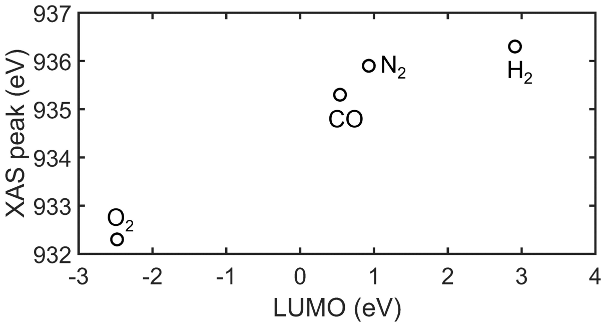

Figure S4: The dependence of the peak position (energy) of the new NEXAFS feature that emerges upon ligand chemisorption as a function of the LUMO energy of the isolated ligand small molecule.

Table S2: Panel A: (Upper rows) The calculated energies and orbital plots of the HOMO and LUMO of the free O_2, CO, N_2, H_2, C_2H_4, and NH_3 molecules, respectively. (Lower rows) The binding energy, experimental XAS peak energy, and the predicted orbital projection (C^2) of the adsorbed molecules. C^2 values were calculated using Equation 6 as given in the main text. The def2-SVPD basis was used for the calculations. The isosurface for the orbital wavefunction plot is 0.05. The Cu 3d orbital is assumed to be −9.9 eV. Panel B: The LUMO+1 to LUMO+4 orbitals of free NH_3.

Panel A

	O_2	CO	N_2	H_2	C_2H_4	NH_3
HOMO (eV)	-10.91	-12.22	-13.93	-13.33	-9.10	-9.17
	π^* 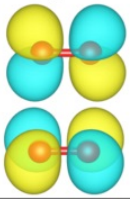	σ 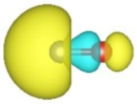	σ 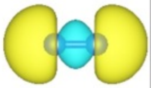	σ 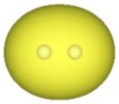	π 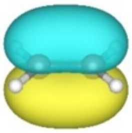	N 2p lone pair 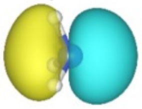
LUMO (eV)	-1.58	0.54	0.95	3.18	1.03	1.52
	π^* 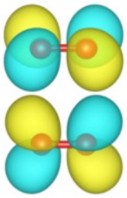	π^* 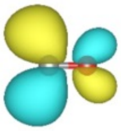	π^* 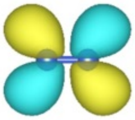	σ^* 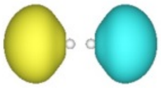	π^* 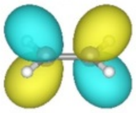	sp^3 σ^* 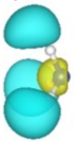
Binding energy (eV)	-0.48	-0.80	-0.43	-0.34	-0.85	-0.95
Exp. peak (eV)	932.3	935.3	935.9	936.3	935.4	
C^2	0.06	0.08	0.04	0.03	0.08	0.08

Panel B

NH_3	Energy (eV)	Orbital plot
LUMO+1	3.61	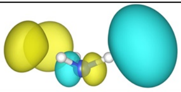
LUMO+2	3.61	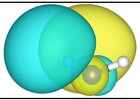
LUMO+3	5.30	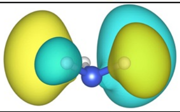
LUMO+4	5.30	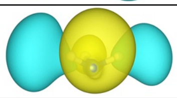

In situ gas dosing NEXAFS spectra

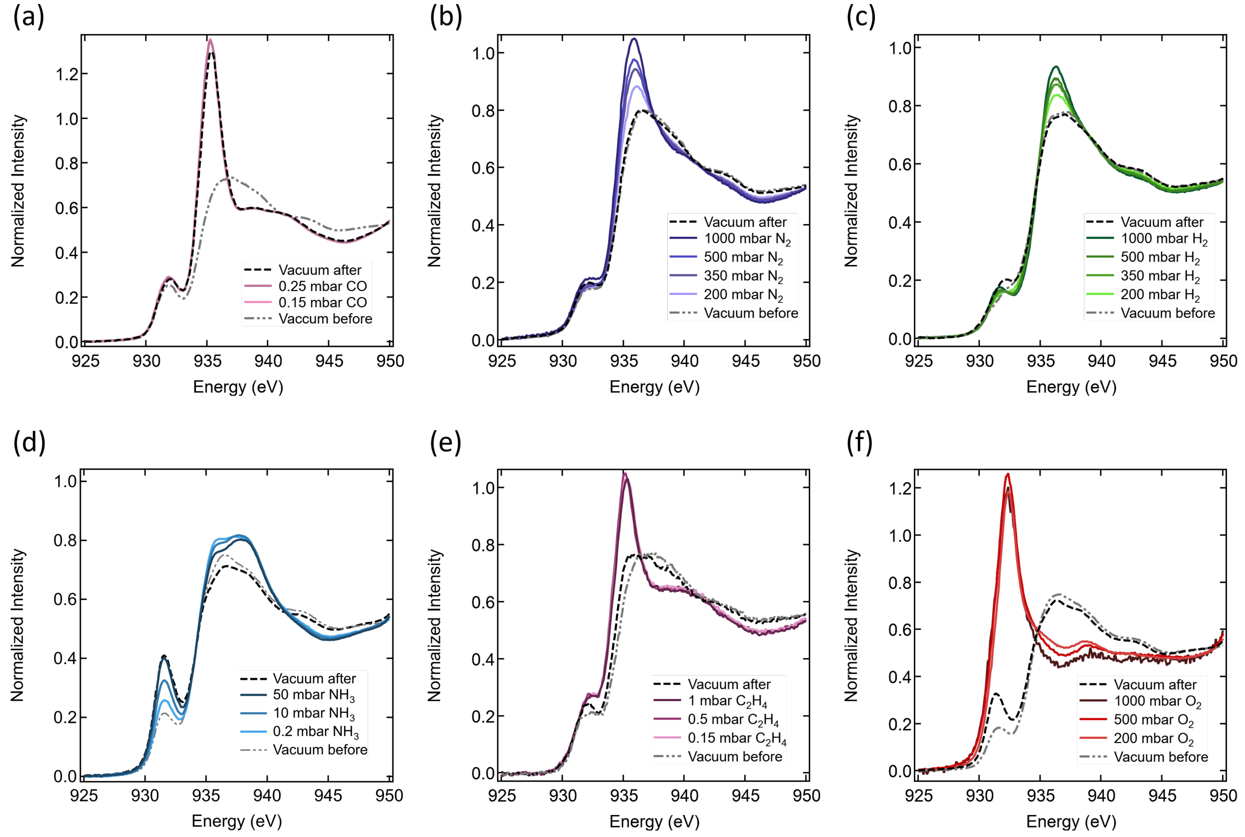

Figure S5: Room temperature *in situ* experimental Cu L-edge NEXAFS data for Cu^{I}-MFU-4*l* dosed with (a) CO, (b) N_2, (c) H_2, (d) NH_3, (e) C_2H_4, or (f) O_2.

Gas adsorption isotherms

Gas adsorption isotherms were collected on a Micromeritics ASAP 2020 or 3Flex gas sorption analyzer. In a typical measurement, fully activated Cu^{I}-MFU-4l (0.05-0.10 g) that had been stored in an argon-filled glovebox was loaded into a glass tube and capped with a Transeal. The tube was connected to the instrument and briefly heated to 180 °C to confirm that no significant off-gassing occurred. The tube was then submersed in a Julabo F-25 circulating isothermal water bath. Free space measurements were performed using UHP-grade He (99.999%) at the analysis temperature. Oil-free vacuum pumps and oil-free pressure regulators were used for all measurements to prevent contamination of the samples during evacuation or of the feed gases during isotherm measurements. All gases used for isotherm measurements were rated as UHP-grade (99.999% purity) with the exception of CO (99.9%) and C_2H_4 (99.90%). Note that the theoretical concentration of open Cu^+ sites based off of the framework molecular formula[S1] is approximately 1.7 mmol/g, although isotherm measurements indicated that samples exhibited approximately 1.4 mmol/g of Cu^+ sites available for chemisorption.

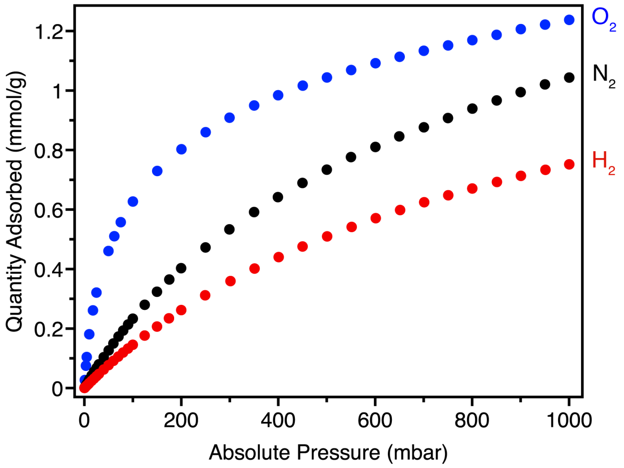

Figure S6: Oxygen (O_2, blue circles), nitrogen (N_2, black circles), and hydrogen (H_2, red circles) isotherms for Cu^{I}-MFU-4l at 25 °C.

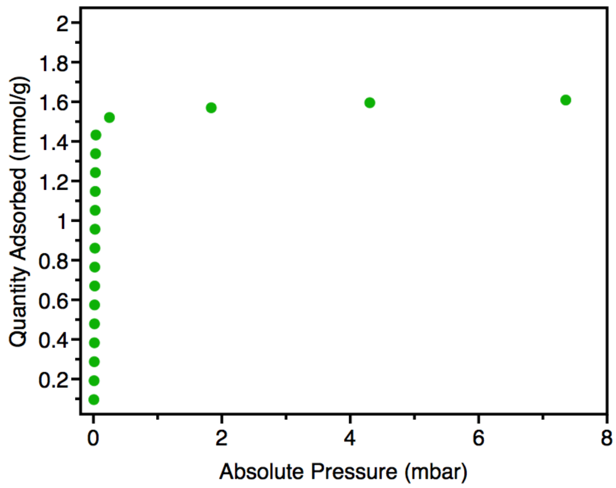

Figure S7: Carbon monoxide (CO) isotherm for the Cu^{I}-MFU-4l framework measured at 25 °C.

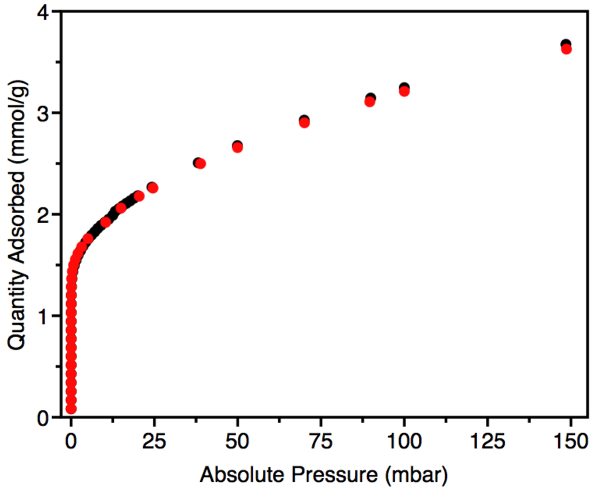

Figure S8: Ammonia (NH_3) isotherms for Cu^{I}-MFU-4l measured between 0-–150 mbar at 25 °C. After the first measurement (black circles), the framework was reactivated at 180 °C *in vacuo* for approximately 6 h. The subsequently-collected NH_3 isotherm (red circles) overlays with the initial data, confirming that the framework is stable under temperature/P_{NH_3} conditions relevant to the reported NEXAFS data collection.

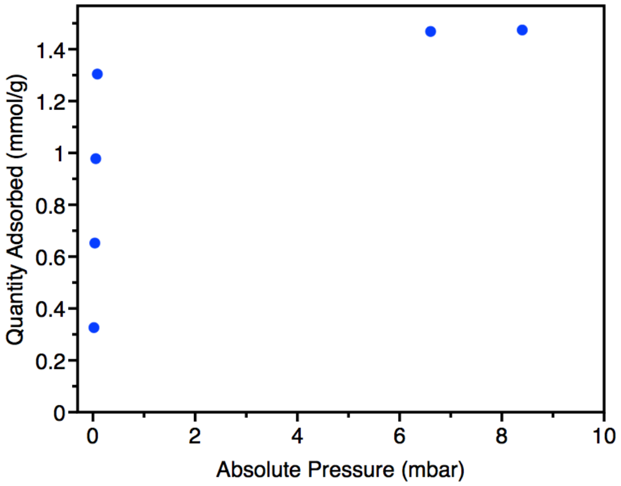

Figure S9: Ethylene (C_2H_4) isotherm for the Cu^I-MFU-4l framework measured at 25 °C.

Correlating peak intensity with binding strength

Molecular orbital energies of the framework-ligand complex can be estimated based on a two-level Hamiltonian model.

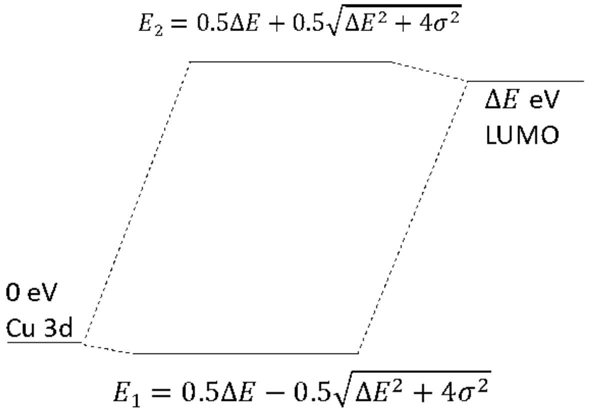

Figure S10: Schematic of the two level model representing the framework and guest molecule interaction.

$$\mathrm{H} = \begin{bmatrix} \Delta E & \sigma \\ \sigma & 0 \end{bmatrix} \begin{matrix} |\mathrm{LUMO}\rangle \\ |\mathrm{Cu_{3d}}\rangle \end{matrix} \tag{1}$$

ΔE: energy difference between Molecular LUMO and Cu 3d orbital

σ: the coupling between molecular LUMO level and Cu 3d orbital

By diagonalizing the Hamiltonian matrix, we have the bonding state:

$$\mathrm{V}_1 = \begin{bmatrix} 0.5\Delta E - 0.5\sqrt{\Delta E^2 + 4\sigma^2} \\ \sigma \end{bmatrix} \tag{2}$$

and $E_1 = 0.5\Delta E - 0.5\sqrt{\Delta E^2 + 4\sigma^2}$

Antibonding state:

$$\mathrm{V}_2 = \begin{bmatrix} 0.5\Delta E + 0.5\sqrt{\Delta E^2 + 4\sigma^2} \\ \sigma \end{bmatrix} \tag{3}$$

$E_2 = 0.5\Delta E + 0.5\sqrt{\Delta E^2 + 4\sigma^2}$

Cu projection in antibonding state:

$$C^2 = \frac{\sigma^2}{\sigma^2 + (0.5\Delta E + 0.5\sqrt{\Delta E^2 + 4\sigma^2})^2} \tag{4}$$

If $\Delta E \gg \sigma$ Then

$$C^2 = \frac{\sigma^2}{\sigma^2 + (0.5\Delta E + 0.5\sqrt{\Delta E^2 + 4\sigma^2})^2} = \frac{\sigma^2}{\Delta E^2 \left(2\frac{\sigma^2}{\Delta E^2} + 0.5 + 0.5\sqrt{1 + \frac{4\sigma^2}{\Delta E^2}}\right)} \approx \frac{\sigma^2}{\Delta E^2} \tag{5}$$

So the coupling strength determines the XAS peak intensity.

Binding energy :

$$E_b = 0.5\sqrt{\Delta E^2 + 4\sigma^2} - 0.5\Delta E = 0.5\Delta E(\sqrt{1 + 4\frac{\sigma^2}{\Delta E^2}} - 1) \approx 0.5\Delta E \left(1 + 2\frac{\sigma^2}{\Delta E^2} - 1\right) \approx \frac{\sigma^2}{\Delta E} \tag{6}$$

Therefore, Cu projection $C^2 \approx \frac{E_b}{\Delta E}$.

This shows that the NEXAFS peak intensity of the MOF–guest complex is proportional to the binding energy. The NEXAFS peak intensity can therefore be used to determine the relative binding energies of different guest molecules, provided that the isolated guest molecules have similar LUMO energies. This observation can be more generally applied to analyze the X-ray absorption spectra of similar MOFs with adsorbed guests.

Moleclar orbital diagram of NH_3-dosed Cu^{I}-MFU-4l

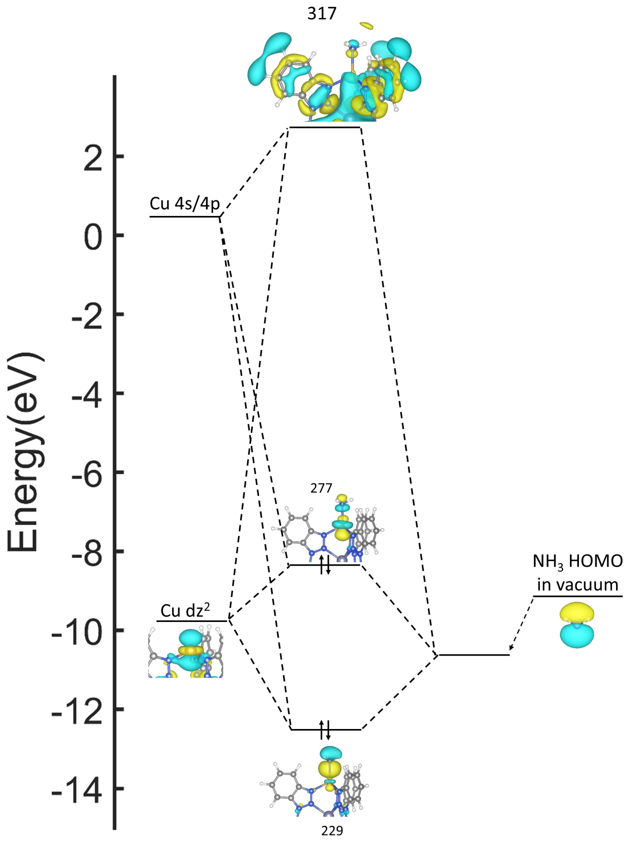

Figure S11: Molecular orbital diagram showing the primary σ donation interactions for NH_3-dosed Cu^{I}-MFU-4l based on DFT calculations. NEXAFS is not sensitive to these interactions, but instead probes weaker backbonding interactions with the NH_3 LUMO+1 and LUMO+2 orbitals, as shown in Figure 3b in the main text.

Natural Bond Orbital charge analysis

Table S3: Calculated copper and ligand atom charges determined from NBO analysis (units of e).

Cu^I-MFU-4*l*	CO-dosed Cu^I-MFU-4*l*	N_2-dosed Cu^I-MFU-4*l*	H_2-dosed Cu^I-MFU-4*l*	C_2H_4-dosed Cu^I-MFU-4*l*	NH_3-dosed Cu^I-MFU-4*l*	O_2-dosed Cu^I-MFU-4*l*
Cu: 0.889	Cu: 0.906	Cu: 0.909	Cu: 0.907	Cu: 0.967	Cu: 0.880	Cu: 1.39
	C: 0.425	N: -0.101	H: -0.008	C: -0.523	N: -1.184	O: -0.302
	O: -0.459	N: 0.087	H: -0.012	C: -0.505	H: 0.401	O: -0.302
				H: 0.231	H: 0.409	
				H: 0.235	H: 0.409	
				H: 0.238		
				H: 0.236		

Orientation and quantum state of O_2-dosed Cu^I-MFU-4*l*

Both singlet and triplet configurations of the O_2 molecule absorbed on the MOF cluster were calculated. The adsorbed O_2 molecule was found to adopt two orientations, a bent end-on orientation (Figure S12a) and a side-on orientation (Figure S12b). The total energy of the triplet side-on and bent end-on O_2 are 1.5 and 1.4 eV lower than the corresponding singlet states, respectively. Therefore we exclude the singlet states as possible configurations. The triplet state side-on O_2 configuration has a lower total energy than the triplet state bent end-on configuration and the corresponding simulated spectrum is a better match to the experimental O_2-dosed spectrum than the triplet end-on configuration (Figure S12c and d). However, the simulated spectrum of the triplet bent end-on configuration features a higher energy peak near 938 eV that may coincide with a weak feature in the experimental spectrum, suggesting a small proportion of O_2 may bind in a bent end-on fashion.

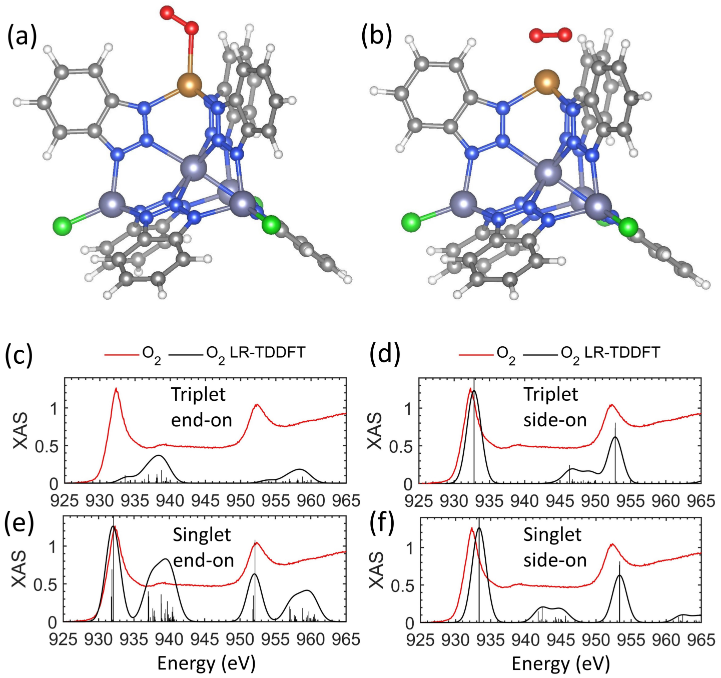

Figure S12: Cu^I-MFU-4*l* with adsorbed O_2 bound in (a) a bent end-on orientation and (b) a side-on orientation. Simulated NEXAFS spectra of triplet (c) and singlet (e) bent end-on O_2 and triplet (d) and singlet (f) side-on O_2 (black traces) along with the experimental O_2-dosed spectrum (red traces). The total energy of the triplet bent end-on structure is 0.23 eV higher than triplet side-on structure.

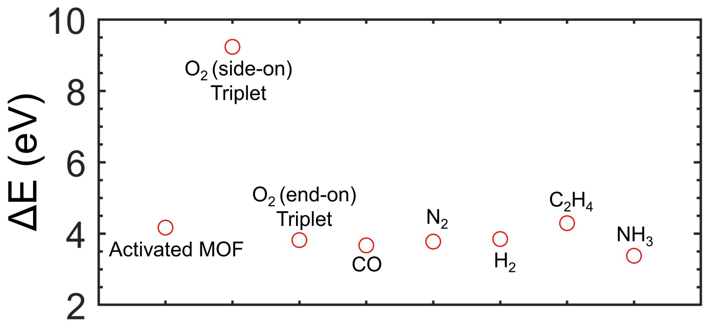

Figure S13: The MOM total energy correction with respect to the first LR-TDDFT root for Cu 2p excitations of Cu^{I}-MFU-4l with various gases (triplet side-on O_2, triplet bent end-on O_2, CO, N_2, H_2, C_2H_4 and NH_3), defined as $\Delta E = E_{MOM} - E_{GS} - \epsilon^{1st\,root}_{LR-TDDFT}$. The most significant correction is applied to the side-on triplet O_2, which would not align with experiment using LR-TDDFT alone.

Effective charge on Cu

The effective charge on Cu is estimated with the total energy when a probe charge is placed near the bare MOF cluster.

The total energy of the system with a MOF cluster and the probe charge is

$$E = \frac{1}{4\pi\epsilon}\frac{q_1 q_2}{r}$$

where $\epsilon = 8.854 \times 10^{12} C \cdot V^{-1} \cdot m^{-1}$ and $e = 1.602 \times 10^{-19} C$. The effective charge on Cu is

$$q_1 = \frac{E \cdot r}{14.4 q_2}$$

The unit of q is elementary charge e, the unit of the energy is eV and the unit of r is Å. When the probe charge is placed on along the Zn–Cu direction and 2 Å above the Cu atom, the calculated effective charge on Cu is 0.2 e.

When the free gas molecules are in close proximity to a positive charge, an electrostatic shift lowers molecular orbital energies. Tables S4-S10 show this electrostatic shift for each gas studied. For all of these calculations, the distance between the point charge and the

molecule is the same as Cu–guest distance based on the relaxed geometry of each respective framework–guest complex.

Table S4: The orbital energies of H_2 in vacuum and in the electrostatic field of +0.2 e, +0.5 e and +1 e point charges. The H_2 geometry is obtained from the relaxed MOF–H_2 structure. The charge is placed at the position of Cu atom.

H_2 orbital energy (eV)				
orbital index	neutral	+0.2e	+0.5e	+1e
1	–13.22	–14.72	–17.01	–20.98
2 LUMO	2.91	1.80	–0.14	–4.71
3	5.03	3.29	0.33	–3.54
4	5.03	3.84	1.82	–1.47
5	6.31	5.14	3.40	0.52
6	8.76	7.40	5.36	2.10
7	8.76	7.62	5.93	3.07

Table S5: The orbital energies of CO in vacuum and in the electrostatic field of +0.2 e, +0.5 e and +1 e point charges. The charge is placed at the position of Cu atom.

CO orbital energy (eV)				
orbital index	neutral	+0.2e	+0.5e	+1e
5	–15.29	–16.49	-18.34	–21.71
6	–15.29	–16.49	–18.34	–21.71
7	–12.22	–13.80	–16.22	–20.65
8 LUMO	0.54	–0.76	–2.83	–7.97
9	0.54	–0.76	–2.83	–6.50
10	2.10	1.12	–1.77	–6.50

Table S6: The orbital energies of C_2H_4 in vacuum and in the electrostatic field of +0.2 e, +0.5 e and +1 e point charges. The charge is placed at the position of Cu atom.

C_2H_4 orbital energy (eV)				
orbital index	neutral	+0.2e	+0.5e	+1e
5	–14.61	–15.76	–17.52	–20.63
6	–13.12	–14.29	–16.08	–19.27
7	–11.37	–12.49	–14.20	–17.22
8	–9.01	–10.34	–12.44	–16.16
9 LUMO	0.87	–0.33	–2.23	–5.55
10	1.41	0.41	–1.39	–5.25

Table S7: The orbital energies of N_2 in vacuum and in the electrostatic field of +0.2 e, +0.5 e and +1 e point charges. The charge is placed at the position of Cu atom.

N_2 orbital energy (eV)				
orbital index	neutral	+0.2e	+0.5e	+1e
4	–17.12	–18.34	–20.27	–23.78
5	–14.78	–15.92	–17.66	–20.82
6	–14.78	–15.92	–17.66	–20.79
7	–13.93	–15.16	–17.01	–20.19
8 LUMO	0.93	–0.24	–2.04	–6.61
9	0.93	–0.24	–2.04	–5.22

Table S8: The orbital energies of NH_3 in vacuum and in the electrostatic field of +0.2 e, +0.5 e and +1 e point charges. The charge is placed at the position of Cu atom.

NH_3 orbital energy (eV)				
orbital index	neutral	+0.2e	+0.5e	+1e
3	–14.42	–15.62	–17.52	–20.93
4	–14.39	–15.59	–17.50	–20.90
5	–9.20	–10.61	–12.84	–16.84
6 LUMO	1.50	0.44	–1.39	–5.47
7	3.62	2.72	1.33	–1.82
8	3.62	2.72	1.33	–1.14

Table S9: The orbital energies of side-on O_2 in vacuum, and in the electrostatic field of +0.2 e, +0.5 e and +1 e point charges. The charge is placed at the position of Cu atom.

O_2 side-on orbital energy (eV)								
	neutral		+0.2e		+0.5e		+1e	
orbital index	up	down	up	down	up	down	up	down
6	–17.82	–14.01	–19.21	–15.51	–21.33	–17.82	–25.03	–21.85
7	–17.36	–14.01	–18.72	–15.40	–20.79	–17.50	–24.41	–21.14
8	–11.81	–2.48 (LUMO)	–13.22	–3.89 (LUMO)	–15.43	–6.07 (LUMO)	–19.24	–9.80(LUMO)
9	–11.78	–2.48	–13.17	–3.81	–15.29	–5.85	–18.97	–9.42
10	1.61	1.74	0.68	0.84	–2.23	–1.80	–8.16	–7.48

Table S10: The orbital energies of bent end-on O_2 in vacuum and in the electrostatic field of +0.2 e, +0.5 e and +1 e point charge. The charge is placed at the position of Cu atom.

O_2 end-on orbital energy (eV)								
	neutral		+0.2e		+0.5e		+1e	
orbital index	up	down	up	down	up	down	up	down
6	–18.64	–14.99	–19.73	–16.11	–21.39	–17.80	–24.25	–20.74
7	–17.82	–14.99	–18.91	–16.08	–20.60	–17.71	–23.48	–20.57
8	–10.94	–1.61(LUMO)	–12.05	–2.72(LUMO)	–13.74	–4.52(LUMO)	–16.68	–8.03(LUMO)
9	–10.94	–1.61	–12.03	–2.67	–13.69	–4.30	–16.57	–7.13
10	1.80	1.93	0.63	0.73	–1.85	–1.80	–6.56	–6.26

References

(S1) FitzGerald, S. A.; Mukasa, D.; Rigdon, K. H.; Zhang, N.; Barnett, B. R. Hydrogen Isotope Separation within the Metal–Organic Framework Cu(I)-MFU-4l. *J. Phys. Chem. C* **2019**, *123*, 30427–30433.
